# Risk factors for bariatric surgery in patients over 65 years of age—a multicenter retrospective cohort study

**DOI:** 10.1007/s00423-024-03304-0

**Published:** 2024-04-09

**Authors:** Natalia Dowgiałło-Gornowicz, Paweł Lech, Bartosz Katkowski, Maciej Walędziak, Monika Proczko-Stepaniak, Michał Szymański, Izabela Karpińska, Piotr Major

**Affiliations:** 1 Department of General, Minimally Invasive and Elderly Surgery, Collegium Medicum, University of Warmia and Mazury, 10-045 Olsztyn, Poland; 2 Department of General and Vascular Surgery, Specialist Medical Center, 57-320 Polanica Zdrój, Poland; 3 Department of General, Oncological, Metabolic and Thoracic Surgery, Military Institute of Medicine, 04-141 Warsaw, Poland; 4 Department of General, Endocrine and Transplant Surgery, Medical University of Gdansk, 80-214 Gdańsk, Poland; 5 2nd Department of General Surgery, Jagiellonian University Medical College, 30-688 Cracow, Poland; 6 Doctoral School of Medical and Health Sciences, Jagiellonian University Medical College, Cracow, Poland

**Keywords:** Metabolic surgery, Bariatric surgery, Older patients, Elderly, Risk factors

## Abstract

**Purpose:**

Societies are aging, life expectancy is increasing, and as a result, the percentage of elderly people in the population is constantly increasing. When qualifying patients over 65 years of age for bariatric surgery, the benefits and risks should be carefully assessed. Weighing risk factors against each other to improve the quality of life and better control of obesity-related diseases. The study aimed to determine risk factors for bariatric surgery among patients over 65 years of age.

**Methods:**

A multicenter, retrospective analysis of patients undergoing laparoscopic bariatric procedures from 2008 to 2022. The patients were divided into two groups: complicated (C) and uncomplicated (UC). Uni- and multivariate logistic regression analysis was performed to obtain significant, independent risk factors.

**Results:**

There were 20 (7.0%) patients in C group and 264 (93.0%) patients in UC group. The most common complication was intraperitoneal bleeding (8, 2.8). There was no postoperative mortality. The mean follow-up was 47.5 months. In a multivariate logistic regression analysis, length of stay and %EWL significantly corresponded to general complications (OR 1.173, OR 1.020). A higher weight loss before surgery lowered the risk for hemorrhagic events after surgery (OR 0.889). A longer length of stay corresponded to leak after surgery (OR 1.175).

**Conclusions:**

Bariatric and metabolic surgery appears to be a safe method of obesity treatment in patients over 65 years of age. The most common complication was intraperitoneal bleeding. A prolonged hospital stay may increase the risk of leakage, while a higher weight loss before the surgery may lower the risk of bleeding.

## Introduction

Societies are aging, life expectancy is increasing, and as a result, the percentage of elderly people in the population is constantly rising [1]. Initially, surgery was thought to be reserved for individuals under the age of 60–65 [2]. However, recently, there have been a few reports of patients operating above this age limit [3–5]. According to the latest guidelines, patients should be assessed individually, considering their actual biological age rather than their chronological age, to assess the benefit-risk ratio before bariatric surgery and make informed decisions about qualification for the procedure [6, 7].

Quillot et al. described the paradox in bariatric surgery [8]. The positive effects of surgery often increase with the risk of surgery, as obesity itself is the cause of multiple surgical risk factors in most cases [8]. The impact of some perioperative and postoperative complications can be reduced before surgery by taking specific actions, such as smoking cessation, weight reduction, compensation of chronic diseases, treatment of obstructive sleep apnea, selection of the appropriate type of bariatric surgery and the technique itself, and involvement of an experienced surgical team [8, 9]. Unfortunately, some risk factors cannot be changed. These include functional impairment, age, cardiovascular disease, and gender [8, 9]. Nevertheless, surgery appears to be the most effective treatment for obesity that can improve quality of life with an acceptable level of risk [10, 11]. The mortality rate after bariatric surgery is at the level of knee arthroplasty, and postoperative complications at the level of laparoscopic cholecystectomy or hysterectomy [12].

When qualifying patients over 65 years of age for surgery, the benefits and risks should be carefully assessed, weighing aforementioned risk factors against each other to improve the quality of life and better control of obesity-related diseases [9, 13]. The study aimed to determine risk factors for bariatric surgery among patients over 65 years of age.

## Materials and methods

It is a multicenter, retrospective cohort study of a collected database of patients undergoing laparoscopic bariatric procedures from 2008 to 2022. The data came from 11 bariatric centers, each performing over 100 surgeries annually. There were 284 patients included in the study. Inclusion criteria were meeting the eligibility criteria for bariatric surgery and being over 65 years of age. Patients with missing or inconsistent data were excluded from the study. The patients were categorized into two groups: complicated (C) and uncomplicated (UC). The analysis is in line with STROCSS guidelines [14].

The database contained demographic characteristics of patients (sex, age, maximal weight, weight before surgery, weight loss before surgery, body mass index (BMI)) and information on type 2 diabetes (T2D) and hypertension (HT) (type and length of treatment). It contained data about complications according to Clavien-Dindo classification, both 30-day and long-term complications. It also included information on the surgery (type of surgery, operative time, length of hospital stay) and outcomes of bariatric treatment (current weight and BMI, percentage of excess weight loss (%EWL), percentage of total weight loss (%TWL), T2D and HT remission). The outcomes of bariatric surgery were described in accordance with ASMBS outcome reporting [15]. All results correspond to the follow-up time.

Surgical techniques and perioperative care protocols, including preoperative, intraoperative, and postoperative interventions, were standardized across all participating centers. Patients were treated by a multidisciplinary team of surgeons, physicians, nurses, nutritionists, and psychologists at each bariatric center.

### Statistical analysis

Descriptive statistical analysis was conducted using Statistica software 13.PL (StatSoft Inc.). Continuous variables were presented as means and standard deviations or medians with interquartile ranges when appropriate. Categorical variables were compared using the chi-square test. Quantitative variables were compared using the *U* Mann–Whitney test. Significant variables in univariate logistic regression models were adjusted in multivariate analysis to obtain significant, independent risk factors and to calculate the odds ratios (OR) with 95% confidence interval (CI). A *p*-value of ≤ 0.05 was considered statistically significant.

## Results

A total of 284 patients over 65 years old underwent bariatric surgery. The patients were divided into two groups: C and UC. 20 patients (7.0%) were in the C group, while 264 (93.0%) were in the UC group. The most common complication was intraperitoneal bleeding (8, 2.8%), Table 1. There were 13 (4.6%) 30-day Clavien Dindo III complications: 8 (2.8%) intraperitoneal bleedings, 3 (1.1%) leaks, and 2 intraabdominal abscesses (0.7%). There was no postoperative mortality. The mean follow-up was 47.5 months ± 32.2 months. The follow-up rate was 78.0%.

**Table 1:** Characterictics of complications

Complication	N, %
Intraperitoneal bleeding	8, 2.8%
Leak	3, 1.1%
Intraperitoneal abscess	2, 0.7%
Thightening of sleeve	3, 1.1%
Bile reflux	2, 0.7%
Mesenteric vein thrombosis	1, 0.4%
Internal hernia	1, 0.4%

Table 2 compares the pre- and postsurgical characteristics of patients between the study groups. Patients from the C and UC groups did not differ statistically in terms of sex, age, BMI before surgery, weight loss before surgery, interventions before surgery, type and duration of treatment of T2D or HT, type of surgery, operative time, actual BMI, TWL%, EWL% and remissions of T2D and HT. Statistically significant difference was observed in the length of hospital stay (*p* = 0.022)

**Table 2:** Characteristics of patients

	C group	UC group	*p*-value
N (%)	20 (7.0%)	264 (93.0%)	
Female/Male, *n* (%)	13/7 (65.0%)	160/104 (60.6%)	0.698
Median age, years (IQR)	66 (66–67)	67 (66–68)	0.723
Median BMI, kg/m^2^ (IQR)	42.8 (40.1–45.7)	42.8 (39.1–46.9)	0.793
Median weight loss before surgery, kg (IQR)	3.5 (0.5–10)	5.0 (1.0–10.0)	0.568
Intragastric balloon before surgery	2	24	0.892
Pharmacotherapy before surgery (GLP-1)	0	8	0.430
T2D, n (%)	11 (55.0%)	135 (51.1%)	0.741
Duration of T2D before surgery*			
< 5 years	2	42	0.728
5–10 years	3	29	
> 10 years	4	43	
HT, n (%)	16 (80.0%)	228 (86.4%)	0.436
Duration of HT before surgery*			
< 5 years	1	20	0.985
5–10 years	3	59	
> 10 years	5	95	
Types of surgery			
AGB	0	6	0.371
SG	15	218	
OAGB	4	23	
RYGB	1	17	
Operative time, min (IQR)	62.5 (52.5–85.0)	64.0 (50.0–90.0)	0.899
Length of stay, days (IQR)	3.9 (1.5–4.0)	2.4 (2.0–3.0)	**0.022**
Actual BMI, kg/m^2^ (IQR)	33.1 (27.4–35.1)	42.8 (39.1–46.9)	0.196
TWL%, % (IQR)	22.0 (18.6–30.3)	20.0 (13.9–26.0)	0.107
EWL%, % (IQR)	52.4 (49.0–86.6)	50.0 (35.2–65.4)	0.065
T2D remission, n			
Complete remission	9	46	0.884
Partial remission	3	39	
Improvement	2	41	
No changes	1	9	
none	9	129	
HT remission, n			
Complete remission	4	51	0.774
Partial remission	2	55	
Improvement	7	91	
No changes	3	31	
none	4	36	

*data not available in all cases

All available risk factors for complications were analyzed in univariate logistic regression models, Table 3. In a multivariate logistic regression analysis, length of stay and %EWL significantly corresponded to general complications (OR 1.173, *p* = 0.024; OR 1.020, *p* = 0.031, respectively). A higher weight loss before surgery lowered the risk for hemorrhagic events after surgery (OR 0.889, *p* = 0.039), Table 4. A longer length of stay corresponded to leaks after surgery (OR 1.175, *p* = 0.015), Table 4.

**Table 3:** Univariate logistic regression analysis for risk factors for complications

Variable	All complications		Bleeding		Leakage	
	OR	*p*	OR	*p*	OR	*p*
Sex,male	0.828	0.698	0.511	0.416	0.777	0.838
Age	1.006	0.969	0.600	0.154	0.471	0.278
T2D	1.168	0.739	0.944	0.936	0.469	0.538
HT	0.632	0.434	1.258	0.071	n/a	n/a
Intragastric balloon before surgery	1.106	0.896	0.139	3.486	0.000	0.997
Pharmacotherapy before surgery	0.000	0.998	0.000	0.998	0.000	0.998
Weight loss before surgery	0.976	0.438	**0.889**	**0.039**	0.931	0.413
BMI	0.998	0.967	0.976	0.704	1.062	0.507
Time before first visit and surgery	1.024	0.359	1.041	0.192	1.048	0.249
Length of stay	**1.116**	**0.045**	1.089	0.221	**1.183**	**0.012**
Operative time	0.994	0.444	0.983	0.263	1.005	0.683
Type of surgery (SG ref.)						
AGB	0.000	0.998	0.000	0.998	0.000	0.999
OAGB	2.528	0.125	1.242	0.842	0.000	0.998
RYGB	0.855	0.882	0.000	0.998	6.794	0.125
Actual BMI	0.936	0.157	0.969	0.652	0.736	0.928
TWL%	1.043	0.074	1.008	0.825	1.062	0.303
EWL%	**1.020**	**0.031**	1.003	O.826	1.026	0.257
T2D remission	1.558	0.447	0.670	0.723	1.360	0.804
HT remission	0.706	0.638	0.228	0.209	0.135	0.872

**Table 4:** Multivariate logistic regression analysis for risk factors for complications

Variable	All complications			Bleeding			Leakage		
	OR	CI	*p*	OR	CI	*p*	OR	CI	*p*
Lenght of stay	1.173	1.021–1.348	0.024	-		-	1.175	1.032–1.339	0.015
%EWL	1.020	1.002–1.039	0.031	-		-	-		-
Weight loss before surgery	-		-	0.889	0.796–0.994	0.039	-		-

## Discussion

Our study is a retrospective analysis of the risk of complication after bariatric and metabolic surgery. The analysis is based on data from 284 patients over 65 years of age. Complications occurred in 7.0% of patients in this age group. To our knowledge, this is one of the largest cohorts of patients over 65 years of age collected as a part of a multidisciplinary long-term follow-up reporting project.

A recent meta-analysis by Vallois et al. demonstrated that the overall morbidity rate was significantly increased in patients over 60 years old compared to younger patients (8.98% vs 6.2% [16]. The occurrence of comorbidities above Clavien-Dindo III was 4.6% in older group and 5.5% in younger group, with no significant difference [16]. In our study, there was 4.6% of major complications, which was in line with this meta-analysis. In the same paper, the authors analyzed the leakage, abscesses, and bleeding rate in patients above 60 years of age. There were 0.6%, 0.47%, and 0.88% respectively and were significantly higher than in younger patients [16]. The authors pointed out that patients over 60 years of age should be judiciously qualified. A Scandinavian study by Stenberg et al. also pointed out that age may be a risk factor for serious complications [17]. An analysis of over 40.000 patients showed that age elevated OR to 1.10, *p* = 0.007.

In our study, we demonstrated a 1.1% leak rate. Two of them occurred after SG, while one occurred after RYGB, with no significant difference. The rate is higher than the one described in the general population. [16, 18]. A large MBSAQIP analysis by Alizadeh et al. demonstrated that age alone is not a risk factor, but HT and T2D are risk factors for leaks (OR 1.36 and OR 1.18, respectively) [17]. Of our group,146 (51.4%) patients suffered from T2D and 244 (85.9%) from HT. Although we did not find a significant relationship between obesity-related diseases and increased risk, the higher-than-population rate of leaks may be due to the multimorbidity of patients.

Elderly patients in our study manifested higher bleeding rate (2.8%) than described in previous population studies [16]. Castro et al. described that HT is associated with higher risk of hemorrhagic complications (OR 5.029) [19]. The higher risk in our group of patients can be explained by the occurrence of HT or taking anticoagulants, despite the lack of statistical significance.

According to our data, the choice of surgery did not affect the occurrence of postoperative complications. The vast majority of complications occurred after SG (75%), which is associated with a significant predominance of these operations in our material (82%). Other authors have observed similar findings [20, 21]. Taking into account the representative group of patients in the meta-analysis, the results are different. Xu et al. reported that the overall complications rate, both early and late, occurred significantly more often after RYGB than SG in elderly patients [22]. The early mortality rate was also higher after RYGB than SG [22]. In a recent study analyzing the MBSAQIP database, Edwards et al. in the multivariate regression model also showed that SG has a lower risk of complications compared to RYGB in elderly patients [9]. Therefore, it is not surprising that surgeons are more likely to choose SG in older patients.

In our study, we showed that the greater the weight loss before surgery, the lower the risk of bleeding complications in the study group of patients. In multicenter study by Stefura et al., the authors demonstrated that unsatisfactory preoperative weight loss in not a risk factor for complications [23]. In the meta-analysis by Roman et al., the authors indicated that preoperative body weight loss has its advantages, but it is also not a decrease in mortality and morbidity [24]. Khan et al. demonstrated that weight loss over 10% 6 months before the surgery may be an independent risk factor for 30-day mortality after bariatric surgery (OR 13.5) [25]. In our opinion, the discrepant results may be related to the method of weight loss and general protein and caloric depletion, which the above studies do not analyze in detail. Therefore, multidisciplinary work on the patient and well-chosen diet before surgery are important [26, 27].

Limitations of the study were its retrospective nature and small sample size. With a small number of patients, we could not draw statistically significant data, only some trends. The conclusions drawn from our research regarding specific types of complications should be interpreted with caution due to the very small number of events. Additionally, some risk factors may introduce bias and be considered confounders; however, they are integral to the overall understanding of long-term patient care. We emphasize that our conclusions are not definitive guidelines for practice but rather serve as observations and encouragement for vigilant monitoring of these patients. Moreover, SG significantly predominated among operations, so observations on operations should also be interpreted with caution. Another limitation was the lack of partial data on outcomes. The obtained results are the endpoint of the follow-up. However, due to the long follow-up time, the results seem to be relevant.

## Conclusions

The most common complication in patients over 65 years of age was intraperitoneal bleeding. Length of stay and %EWL significantly corresponded to general complications (OR 1.173, OR 1.020, respectively). The higher weight loss before surgery lowered the risk for hemorrhagic events after surgery (OR 0.889). Moreover, longer length of stay corresponded to leaks after surgery (OR 1.175). The above conclusions are not guidelines; they merely provide guidance for managing patients in this patient group and serve as a basis for conducting further extensive research on this topic.
